# Silver Nanocomposites with Enhanced Shelf-Life for Fruit and Vegetable Preservation: Mechanisms, Advances, and Prospects

**DOI:** 10.3390/nano14151244

**Published:** 2024-07-24

**Authors:** Xin Ding, Huan Lin, Jie Zhou, Zhihao Lin, Yanyan Huang, Ge Chen, Yanguo Zhang, Jun Lv, Jing Chen, Guangyang Liu, Xiaomin Xu, Donghui Xu

**Affiliations:** 1 State Key Laboratory of Vegetable Biobreeding, Institute of Vegetables and Flowers, Chinese Academy of Agricultural Sciences, Key Laboratory of Vegetables Quality and Safety Control, Ministry of Agriculture and Rural Affairs of China, Beijing 100081, China; 2 National Center of Technology Innovation for Comprehensive Utilization of Saline-Alkali Land, Dongying 257347, China

**Keywords:** silver nanocomposites, Ag-MOF, shelf-life, preservation mechanism, fruit and vegetable preservation

## Abstract

Reducing fruit and vegetable waste and maintaining quality has become challenging for everyone. Nanotechnology is a new and intriguing technology that is currently being implemented in fruit and vegetable preservation. Silver nanomaterials provide superior antibacterial qualities, biodegradability, and biocompatibility, which expands their potential applications in fruit and vegetable preservation. Silver nanomaterials include silver nanocomposites and Ag-MOF, of which silver nanocomposites are mainly composed of silver nanoparticles. Notably, not all kinds of silver nanoparticles utilized in the preservation of fruits and vegetables are thoroughly described. Therefore, the synthesis, mechanism of action, and advancements in research on silver nanocomposites for fruit and vegetable preservation were discussed in this study.

## 1. Introduction

**Figure 1:**
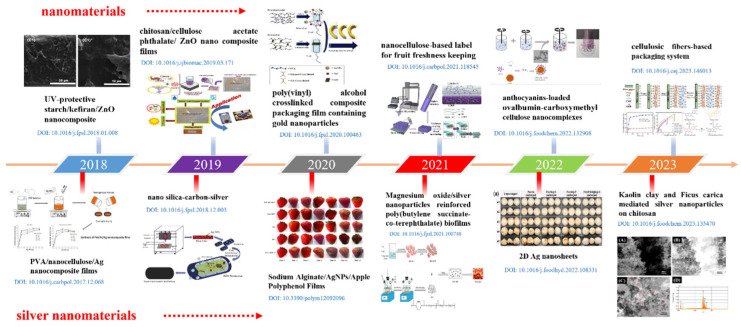
The timeline of nanomaterials and silver nanomaterials in the field of fruit and vegetable preservation. Reprinted with permission from Refs. [15,16,17,18,19,20,21,22,23,24,25,26].

There is an urgent need to promote sustainable fruit and vegetable preservation because food waste amounts to 12 million tons of fruits and 21 million tons of vegetables each year [1]. Preserving fruits and vegetables is essential for decreasing food waste, maintaining food safety, and raising food quality [2,3]. There are many traditional methods of preserving fruits and vegetables that are widely used, such as plastic packaging, low-temperature refrigeration, gas regulation, radiation sterilization, and high-pressure treatment. These conventional techniques, however, may have adverse effects on the environment and public health due to their excessive energy consumption and inadequate preservation capacities [4]. Therefore, the ability to handle these issues using nanomaterials with improved characteristics is a current research priority area [5,6]. In recent years, nanomaterials have been increasingly used in the field of fruit and vegetable preservation (see Figure 1), with the advantages of degradability, effective antibacterial properties, and long-lasting freshness preservation [7,8]. The term “nanomaterials” refers to materials that possess at least one dimension on the nanometer scale (1–100 nm) in three-dimensional space. Currently, organic nanomaterials (e.g., cellulose nanoparticles, chitosan nanoparticles, and starch nanoparticles) and inorganic nanomaterials (e.g., silver nanoparticles, zinc oxide nanoparticles, titanium dioxide nanoparticles, etc.) are applied to the preservation of foods [9]. Because of their exceptional antibacterial qualities, thermal stability, and low toxicity, silver nanocomposites are preferred for food preservation [10].

Silver nanocomposites include nanoscale silver particles or silver nanostructures, which can be categorized into silver-based metal-organic frameworks (Ag-MOFs) and silver nanocomposites (Ag@composites) based on their composition. By releasing silver ions, silver nanocomposites effectively destroy a wide variety of bacteria, fungi, and viruses. It is generally safe to utilize silver ions for fruit and vegetable preservation since they have fewer harmful side effects on human health. Thus, when it comes to fruit and vegetable preservation, silver nanocomposites have more notable antibacterial and environmental qualities than other nanomaterials. They can prolong the shelf life of fruits and vegetables by being used as coating and packaging materials for fruits and vegetables. Currently, a large number of reviews have been published on the application of silver-related nanomaterials in the field of preservation. For example, Shujat et al. [11] summarized the application of phytochemical-mediated silver nanoparticles (Ag NPs) in food packaging and the antimicrobial mechanism. This review focuses on the mechanistic characteristics, preparation-related variables, and nanotechnological aspects of phytochemical-mediated Ag NPs. Similarly, some researchers have reviewed the biosynthesis pathways of Ag NPs and the existing biosynthesis research results [12]. Its potential applications in biosensing, food packaging and preservation, and biomedicine have also been presented. Trotta et al. [13] summarized the application of Ag@composites in food packaging materials, which focuses on the combination of Ag NPs with agar and/or polylactic acid. Furthermore, biopolymers can be characterized as active materials for packaging since they can carry a variety of active compounds. Some researchers provide developments on the use of silver nanoparticles in biopolymer-based packaging [14].

However, most of these published reviews focus only on the synthesis of specific types of silver nanomaterials or their applications. Furthermore, there is no review of the application of various kinds of silver nanocomposites in fruit and vegetable preservation. Therefore, the purpose of this review is to provide a reference for the use of almost all types of silver nanocomposites in fruit and vegetable preservation by introducing the preparation process, preservation mechanism, and recent progress in this area. It also discusses the challenges facing current research and the direction for future development.

## 2. Preparation of Silver Nanocomposites

### 2.1. Preparation of Ag@composites

Aggregation and oxidation of Ag NPs affect their stability and antimicrobial properties. Ag NPs may be mixed with other materials, including polyvinyl alcohol (PVA), proteins, chitosan (CS), TiO_2_, and MgO, to create Ag@composites with many functions, which can help address such problems [27]. By modifying factors including the kind, morphology, and composition of the composite components, the Ag@composites’ particle size, shape, surface properties, and other features may be precisely adjusted. Furthermore, Ag@composites acquired the flexibility, hardness, and other characteristics of other materials in addition to inheriting the features of Ag NPs. As a result, there are more opportunities to use them in the field of fruit and vegetable preservation. Broadly, the modification of Ag@composites can be classified into two methods. In the first method, other materials are added during the synthesis of Ag NPs, which can create composites at the same time. In the second method, Ag NPs are first synthesized and then formed into composites with other materials.

When the target material and silver ions are added to the reaction system simultaneously, they interact with the resulting Ag NPs. The performance of the silver monomer is enhanced by the intimate combination of silver with other components. To create the antimicrobial composite material Ag/polyaniline, Waqas et al. [28] employed a one-pot chemical reduction synthesis method, refluxing silver nitrate and PANI in ethylene glycol at 80 °C for 30 min. PANI can disrupt the structure of cell membranes so that the antimicrobial effect of the composite material is enhanced. Sourav Kumar et al. [29] used the sol-gel method to filter TiO_2_ powder and mix it with silver nitrate for a whole day. After allowing the mixture to dry at 120 °C, it was calcined for 24 h at 600 °C. Ag/TiO_2_ was finally obtained, which is a composite material that can enhance the water resistance and antibacterial qualities of food packaging. In addition, Ag NPs/montmorillonite (MMT) composites were effectively made by combining silver nitrate and MMT and agitating them for a whole night before adding NaBH_4_, then stirring for an additional 12 h. The antibacterial properties of Ag NPs were improved [30]. There is a procedure that produces ring-shaped Au-Ag NPs by stirring HAuCl_4_ and silver nitrate for 30 s before adding ascorbic acid and polyvinylpyrrolidone. This changed the hue of the solution to blue-black, resulting in the formation of porous, spiky Au-Ag NPs. They next mixed the porous, spiky Au-Ag NPs with PVP to form a homogenous and stable colloidal solution, which was heated in a muffle furnace for 12 h at 100 °C [31]. In addition to reacting with a single material to form Ag@composites, Ag NPs can react with two or more materials. After agitating MgO/Ag NPs and silver nitrate in ethanol for 12 h, researchers combined them with polybutylene succinate (PBST) in chloroform to create PBST/MgO/Ag composite films that were more effective in blocking UV rays and exhibiting antibacterial qualities, which were intended for use in cherry tomato packaging [15]. In a similar study, Zhang et al. [32] prepared food cling films with good antimicrobial properties, mechanical properties, photothermal properties, and water vapor barrier by mixing FeCl_3_·6H_2_O and tannic acid (TA), adding silver nitrate, and using a reducing agent to promote the formation of Ag/TA-Fe^3+^. Subsequently, it is mixed with a film-forming solution made of PVA, gels, and CS. Hence, multi-component composites with more comprehensive properties and a wider range of applications can be obtained when silver nanoparticles are compounded with two or more materials.

Ag NPs first were obtained by chemical reduction, biosynthesis, and physical synthesis. They were joined to other materials chemically or mechanically following functionalization or surface modification. This made it possible to control the composition and characteristics of Ag@composites and aided in the creation of oriented composites [33]. Silver nitrate was made to reduce spherical Ag NPs to 13 nm and 9 nm spherical on the surface of diatoms, which can be used as nanoplasma biosensors. The researchers used a photoreduction method that involved irradiating with light at wavelengths of 440 nm and 540 nm, the reducing agent sodium borohydride, and 75 μL of silver nitrate (0.1%) injected every 5 min with continuous stirring. This process was repeated for one hour [34].

The chemical reduction method has a faster reaction rate and is appropriate for large-scale synthesis. However, it has drawbacks such as environmental pollution, difficulty controlling the homogeneity of the dimensions, and the aggregation phenomenon. Depending on the need to regulate the surface properties and stability of Ag NPs, different reducing agents and stabilizers can be used. With the increased awareness of environmental protection, the “green” biosynthesis of Ag NPs has received greater attention. The use of plant-derived components, such as stems, roots, fruits, and leaves, for the synthesis of nanoparticles is preferred over the use of microbial synthesis [35,36]. Bushra Hafeez et al. [37] used potato peel and cilantro stem extracts as reducing agents and placed under light-avoidance conditions for 24 h to prepare Ag NPs with sizes of 65 nm and 70 nm, respectively. The concentration of total flavone and total phenol in both Ag NPs was high. The results indicated that the synthesis of silver nanoparticles from plant extracts could improve their antioxidant properties and biological activities.

Multi-component composites have richer characteristics and can have many properties at once compared to single materials, including antimicrobial, antioxidant, and water-insulating properties. Hassan et al. [38] first synthesized Ag NPs using biosynthesis, then prepared TiO_2_ NPs using the sol-gel method, and finally added them to a mixture of PVA and sodium alginate (SA) for a reaction. They were successful in synthesizing PVA/SA/Ag-TiO_2_ NPs, whose composites significantly enhanced the thermal stability and antimicrobial activity of PVA/SA. Using the direct current sputtering technique, Xiao Ming et al. deposited silver on polymethyl methacrylate (PMMA) with thicknesses of 100 nm and 300 nm [39]. Ag/PMMA films with water resistance and antimicrobial qualities were effectively created.

### 2.2. Preparation of Ag-MOFs

MOFs are crystal-structured materials consisting of metal ions or metal clusters connected with organic ligands through coordination bonds. They have a broad specific surface area, a highly controllable pore structure, a variety of chemical functions, and good stability [40]. Ag-MOF is a kind of silver metal-organic framework that is composed of silver ions and organic ligands. The bacteriostatic impact of silver ions can be enhanced by forming a range of various structures through the use of different organic ligands for coordination. Thus, in the field of preservation, the application of Ag-MOF has received wide attention. The commonly used organic ligands for the preparation of Ag-MOF include imidazoles and carboxylic acids, and the coordination between these organic ligands and silver ions forms a stable lattice structure, which imparts specific chemical and physical properties to the Ag-MOF [41]. Ag-MOF has been created using a variety of techniques, the most widely utilized being mechanochemical synthesis, chemical ultrasound-assisted synthesis, and one-pot synthesis.

**Figure 2:**
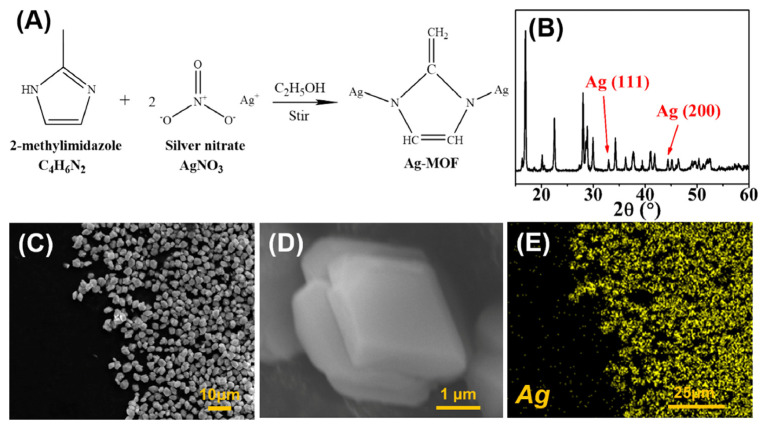
(**A**) The chemical reaction to synthesize Ag-MOFs particles; (**B**) XRD spectrum of Ag-MOF particles; (**C**,**D**) FESEM images of as-prepared Ag-MOF particles with different magnifications; (**E**) silver mapping of Ag-MOFs using EDX. Reprinted with permission from Ref. [42].

**Figure 3:**
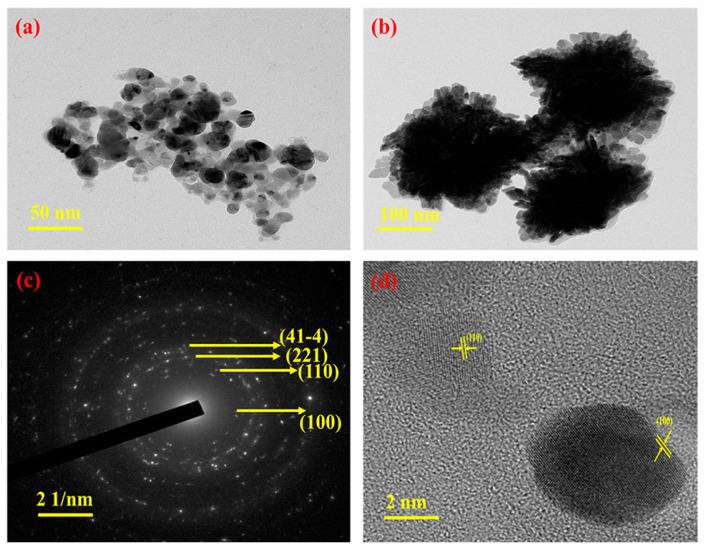
(**a**,**b**) TEM images at different magnifications; (**c**) SAED pattern and (**d**) HRTEM image of the Ag-MOF. Reprinted with permission from Ref. [43].

The one-pot synthesis approach mixes organic ligands, silver salts, and solvents in a reactor for a one-step reaction. It encompasses ambient synthesis, solvothermal synthesis, and hydrothermal synthesis. The morphology, pore structure, and surface characteristics of Ag-MOFs may be controlled by varying factors such as reaction conditions and ligand structure. Yuan et al. [42] dissolved 2-methylimidazole as a ligand in anhydrous ethanol and utilized silver nitrate dissolved in deionized water to provide silver ions, and Ag-MOF was obtained by fully reacting 2-methylimidazole and silver nitrate in a molar ratio of 4:1 for 30 min at room temperature. The X-ray diffraction analysis (XRD) of Ag-MOF is shown in Figure 2, which proves that the Ag-MOF has two typical diffraction peaks of Ag (111) and Ag (200) with a size of about 3.0 μm and a tetrahedral morphology. Shalini et al. [43] used N, N-dimethylformamide (DMF) as the solvent, and terephthalic acid with a molar ratio of 1:1 was stirred with silver nitrate at room temperature for 30 min. Then the temperature was gently raised to 150 °C, kept at 150 °C for 1 h, and then naturally dropped down to room temperature. It was possible to produce a brown, inhomogeneous, spherical Ag-MOF with a specific surface area of 97.325 m^2^g^−1^ (Figure 3). Thus, Ag-MOFs with different morphologies can be synthesized by controlling the ligand, solvent, and reaction temperature. Ag-MOF-5 with high purity was obtained by Song et al. [44] by reacting silver nitrate and terephthalic acid in DMF at 120 °C for 18 h using the solvothermal technique. Moreover, Ag-MOF production may be enhanced by the hydrothermal synthesis process’s high-temperature, high-pressure aqueous environment. Tan et al. [45] stirred silver nitrate and terephthalic acid in DMF for 30 min under light-avoidance conditions and subsequently placed them in a polytetrafluoroethylene autoclave heated at 140 °C for 10 h. When Ag-MOF was finally synthesized, FT-IR analysis revealed Ag-O stretching vibrations, demonstrating that the coordination of the silver nitrate and terephthalic acid was effective. Ag-MOF also displayed an irregular spherical octahedral shape, with a particle size of around 439 nm. The hydrothermal one-pot synthesis is capable of synthesizing not only spherical structures of Ag-MOF but also forming microcluster structures. Meshari et al. [46] dissolved silver nitrate in deionized water and 2-methylimidazole in ammonium hydroxide and heated the mixture at 100 °C for 24 h in a polytetrafluoroethylene autoclave. Ag-MOF particle microclusters with a specific surface area of 676.06 m^2^g^−1^ were obtained. The one-pot synthesis approach for Ag-MOF offers simplicity, efficiency, and tunability. It may, however, require specialized tools and safety precautions due to its sensitivity to reaction conditions and complicated reaction mechanism.

**Figure 4:**
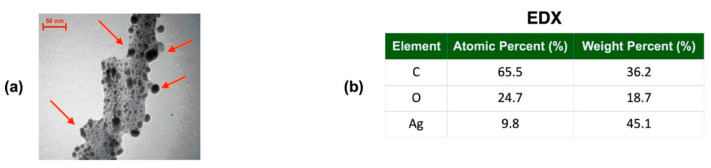
(**a**) TEM micrograph of Ag-MOF nanocrystals; (**b**) element concentration determined via EDX analysis. Reprinted with permission from Ref. [47].

Chemical ultrasound-assisted synthesis is the use of ultrasonic radiation energy to promote the formation of Ag-MOF. High-frequency, high-intensity mechanical vibration is produced in the reaction system by the introduction of ultrasound. This vibration accelerates the rate of reaction, raises yield, and enhances product quality, in addition to causing localized changes in temperature and pressure. Zirehpour et al. [47] obtained Ag-MOF with an average particle size of 33 nm (Figure 4a) by reacting trimesic acid as a ligand with a silver nitrate solution in DMF under ultrasonic waves at a frequency of 24 kHz for 60 min. The three elements, carbon, oxygen, and nitrogen, in the MOF were characterized by EDX analysis, as shown in Figure 4b. Ag-MOF in the desired form can also be synthesized by changing the reaction conditions with no change in the metal ion solution or ligand. Medha et al. [48] reacted silver nitrate mixed with homobenzoic acid at a frequency of 20 kHz, a pulse of 0.6, and an output power of 100 w for 30 min to synthesize Ag-MOF in the form of a cube with a specific surface area of 86 m^2^g^−1^, a pore size of 0.0824 cm^3^g^−1^, and a size of 5.13 nm. This method can rapidly prepare high-quality Ag-MOF materials in a short period of time. Nevertheless, the ultrasonic energy might lead to pyrolysis or material deterioration, potentially impacting material quality. The use of ultrasonography also raises the cost of equipment and complicates operations.

The process of mechanochemically synthesizing Ag-MOF entails mechanically stirring, grinding, or ball milling silver ions and organic ligands in a solvent. For example, Zhao et al. [49] synthesized Ag-MOF with a particle size of 2.0 nm by a milling method that utilizes trimesic acid as a milling solution and mechanical force to pulverize and mix the material. This approach offers various advantages, such as easy handling, flexibility in adding material, and the fact that the reaction may be run at ambient temperature. But it requires a significant amount of mechanical energy, which might cause Ag-MOFs structure and properties to change.

## 3. Preservation Mechanism of Silver Nanocomposites

Nanomaterials freshness in fruits and vegetables through their unique chemical and physical properties, such as antimicrobial effects, antioxidant properties, barrier protection, and climate control [50]. Silver nanocomposites enhance freshness mainly through antimicrobial effects, including synergistic antimicrobial effects with other materials.

Silver nanocomposites may release silver ions too quickly due to the high surface area-to-volume ratio, resulting in toxicity [51]. The mechanism of toxicity is unclear, but proposed mechanisms include oxidative stress and endoplasmic reticulum stress [51]. The safety of silver nanoparticles was studied in albino rats [52], and the liver and kidney-related indexes of mice were tested. The results showed that a certain concentration of Ag NPs was safe. Studies were conducted on the blood compatibility and cytocompatibility of silver nanocomposites used in food preservation [53]. The hemolysis rate and cell survival rate both showed very low levels of cellular toxicity. In addition, the safety limit of silver declared by EU safety regulations for foods was 50.00 μg/L [54]. Nevertheless, if an adequate amount of silver nanocomposite is added to the fresh-keeping material, it will not exceed the safety standard limit of silver ions in food. Yin et al. [54] combined silver nanoparticles with graphene oxide (GO) to form a composite nanomaterial, which was applied to strawberry preservation. The content of silver ions in strawberries was measured after seven days of fresh storage. The silver ions in strawberries were 2.09 μg/L, which is far below the EU food safety standard for silver ions. Therefore, silver ions and Ag NPs could be used as synergistic antimicrobials for bacteriostatic applications.

### 3.1. Ag NPs and Silver Ion Migration

**Figure 5:**
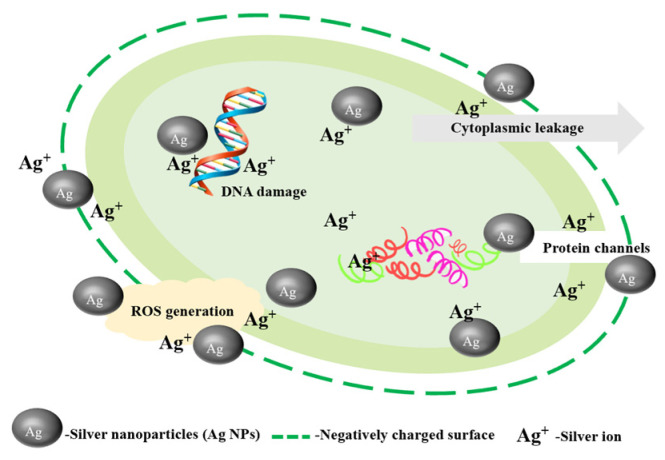
The mechanism involved in the bactericidal activity of silver nanoparticles (Ag NPs) due to their nano and ionic properties.

The antimicrobial mechanism of Ag NPs and silver ions involves several aspects. Ag NPs themselves have strong antimicrobial activity, and they can enter the cytoplasm in two ways: through protein channels and through stomata in the microbial cell wall [55]. The antimicrobial process of Ag NPs is not only due to their direct penetration into the cytoplasm but also involves their conversion to silver ion action, as shown in Figure 5. Silver ions are capable of disrupting negatively charged cell walls, which in turn causes leakage of cellular contents and disrupts cellular integrity [56]. Silver has long been recognized as an antibacterial and antifungal agent against a variety of bacteria and fungi. Silver ions disrupt bacterial metabolism and function by interacting with proteins and peptidoglycan on the bacterial surface. The inhibition of bacterial growth and replication results from the effects of this interaction on cellular respiration, bacterial enzyme activity, and other crucial biological processes [57]. The antifungal characteristics of synthetic Ag NPs against *Alternaria* and *Botrytis* were demonstrated in a study by Ouda, where Ag NPs significantly impacted the breakdown of lipids, proteins, carbohydrates, and acetylglucosamines found in fungal cell walls [58]. Moreover, the biosynthesized Ag NPs exhibited strong antifungal activity against the phytopathogenic fungi *Fusarium oxysporum*, *Alternaria alternata*, *Sclerotinia sclerotiorum*, *Macrophomina phaseolina*, *Rhizoctonia solani*, *Botrytis cinerea*, and so on [59]. Ag-MOF can achieve an antimicrobial effect by releasing silver ions [60]. Pejman et al. [61] synthesized Ag-MOF in situ on ultrafiltration membranes and used Ag-MOF to release silver ions to make the composite membranes have long-lasting antibacterial effects against *Escherichia coli* and *Staphylococcus aureus*. Furthermore, the bacterial cell’s interior may be reached by silver ions and Ag NPs, which can then react with the DNA of the bacterium, leading to the denaturation and destruction of the DNA. It may cause harm to the genetic material of the bacteria and further impede its regular replication and biosynthesis processes [11,62]. Ag NPs prepared by Verduzco-Chavira et al. using chemical reduction and biosynthesis showed inhibitory effects against both *S. aureus* and *Pseudomonas aeruginosa* [63]. Fouad et al. [64] biosynthesized Ag NPs using turmeric and showed significant antimicrobial activity against *Salmonella*. Saheed Ademola et al. [65] utilized pectin to biosynthesize Ag NPs, and pectin with the function of delivering Ag NPs would adhere to the bacterial membrane to enhance the antimicrobial properties. According to their findings, Ag NPs significantly reduced the growth of *E. coli* by acting as bactericidal agents.

### 3.2. Synergistic Antimicrobials in Composites

The antimicrobial effect of Ag@composites is a synergistic effect of multiple components, including the antimicrobial effect of multiple materials as well as photodynamic antimicrobials.

While the silver ions in the composite material inhibit bacterial growth and reproduction, other components may also have complementary antimicrobial properties. Yang et al. [66] stabilized Ag NPs in the spatial network structure of lignin nanoparticles using an in situ reduction. Additionally, they used charge neutralization effects and the phenolic groups in lignin to synergize with the silver ions to enhance their antibacterial properties, thereby inhibiting *S. aureus* and *E. coli*. Alireza et al. [67] synthesized Ag@ZIF-7 using ZIF-7-loaded Ag NPs. The zinc and silver ions easily permeated the cell membranes of many bacteria and acted as a synergistic bactericidal agent by interfering with DNA function and enzyme activities.

**Figure 6:**
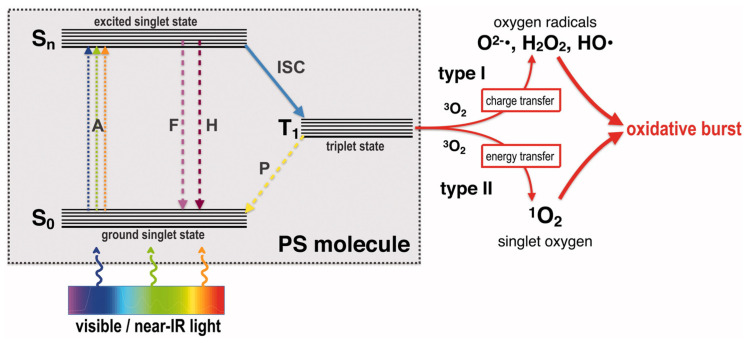
Adapted Jablonski diagram showing the photochemical and photophysical mechanisms of antimicrobial photodynamic therapy (aPDT). S_0_: ground singlet state of the PS molecule; S_n_: excited singlet state of the PS molecule; T1: triplet excited state of the PS molecule; A: absorption of light; F: fluorescence emission; H: heat generation (internal conversion); ISC: inter-system crossing; P: phosphorescence emission; ^3^O_2_: ground state oxygen; ^1^O_2_: singlet oxygen; O^2−•^: superoxide anion; HO•: hydroxyl radical; H_2_O_2_: hydrogen peroxide. Reprinted with permission from Ref. [72].

Singh et al. [68] prepared silver nanosilver/single-walled carbon nanotubes/polypyrrole (Ag/SWCNT/PPy) composites. In addition to the antimicrobial effect of silver ions, SWCNT can trap bacteria inside to form bacteria-CNT aggregates. SWCNT attacks trapped bacteria, causing the bacterial cell membrane to rupture, leading to bacterial death. The data show that the ternary nanocomposites have strong inhibitory effects on *P. aeruginosa* and *E. coli.* Using photosensitizers in the presence of oxygen and a light source with a particular wavelength to generate extremely damaging reactive oxygen species (ROS) is known as photodynamic antimicrobial therapy. ROS have the ability to disrupt the molecular structures of proteins, nucleic acids, and enzymes, as well as the membrane structure of bacteria and other microorganisms. They have the advantages of high safety and high efficiency, and the basic principle is shown in Figure 6 [69]. Liu et al. [70] used curcumin as a photosensitizer, chitosan as a matrix, and silver nanoparticles loaded with curcumin to prepare a composite film, which oxidized the cell membranes and cell walls of bacteria with a large amount of ROS generated by treating the film with blue light, thus causing bacterial damage or death. Ren et al. [71] used CS-loaded silver nanoparticles and methylene blue (MB) as a photosensitizer. Under light conditions, MB in the excited state induces a free radical reaction that converts molecular oxygen to single linear oxygen, while the strong oxidizing property of oxygen radicals converts Ag NPs to silver ions and releases them in solution. The results showed that the composites inhibited the growth of both Gram–negative and Gram–positive bacteria. The advantages of the synergistic impact of the nanosilver composite and photodynamic antibacterial agent include low toxicity, a wide spectrum, and high efficiency. This enhances freshness and offers a workable and trustworthy antibacterial approach for keeping fruits and vegetables fresh.

## 4. Silver Nanocomposites for Freshness Preservation Applications

Currently, silver-related nanomaterials applied to the preservation of fruits and vegetables include Ag NPs and Ag-MOFs. Ag NPs have been extensively applied to develop preservation due to their low toxicity, higher thermal stability, and potent antibacterial activities [73]. However, under conditions of light or high temperature, Ag NPs may aggregate or lose activity. Ag NPs can be combined with other active substances to enhance the effects of fruit and vegetable preservation, such as barrier properties, UV resistance, and antioxidant properties. In addition, MOFs are often utilized in food packaging. Ag-MOFs have the advantages of controllability, structural space diversity, strong adsorption/desorption ability, and so on. Nevertheless, its performance in practical applications may be limited by the complexity and stability of the structure. Bioactive substances that can stabilize Ag-MOF are widely used in fruit and vegetable preservation.

### 4.1. Antimicrobial Activity

The main reason for the deterioration of fruits and vegetables is the action of microorganisms. Therefore, antimicrobial performance is a necessary index to reflect the preservation effect [74]. Ag NPs are interesting because they have a broad range of anti-bacterial and anti-fungal properties [75,76]. Ag NPs can be used as a coating solution to preserve tomatoes [77], and an Ag NPs solution was used for the bacteriostatic experiments on *Penicillium expansum*, *Penicillium italicum*, and *Botrytis cinereus*. It not only had a good inhibitory impact on fungus but also increased tomato shelf life to 17 days. To further enhance the preservative effect, Ag NPs are often synergized with other materials to form films, coated liquids, and granules in a variety of applications [54]. Currently, Ag NPs are more commonly used in the field of preservation in the form of films, which can be embedded in packaging films to achieve a long-lasting antimicrobial effect through the release of Ag NPs from the film. Hassan et al. [38] added TiO_2_ NPs and Ag NPs to PVA/SA and successfully prepared PVA/SA composite films with bimetallic particles by the solution casting method. An SEM image showed that there were uniformly distributed near-spherical Ag-TiO_2_ NPs on the surface of the polymer matrix, and most of them were smaller than 100 nm in size. Thermogravimetric (TGA) measurement revealed that the membrane containing nanoparticles had improved thermal stability and boosted the antibacterial effect when compared to the pure PVA/SA membrane. Additionally, its inhibition of *E. coli* and *P. aeruginosa* would be enhanced with the increase in the proportion of Ag-TiO_2_ NPs in PVA/SA. In order to promote longer-lasting food preservation, this synergistic preservation technique may fully use the antibacterial characteristics of silver nanoparticles in a complementary combination with other materials. When silver ions are mixed directly with the substrate, their antibacterial activity is released throughout the whole surface of the material because the silver ions are distributed evenly throughout the substrate [78]. For example, Martinez-Abad et al. [79] prepared silver-based antimicrobial preservation films by incorporating silver nitrate into ethylene-vinyl alcohol copolymers and performed preservation experiments on food products such as apples. Nonetheless, food safety and human health may be at risk due to the elevated concentration of silver ions. Furthermore, the antimicrobial effect of silver ions in fruits and vegetables with a long shelf life can diminish over time, reducing the freshness preservation effect [76,80].

Ag-MOFs have a large surface area, high porosity, a tunable structure, and long-lasting bactericidal activity through the slow release of silver ions [61,81]. Zhang et al. [82] prepared Ag-MOF with 2-methylimidazole as a ligand using the one-pot method at room temperature and designed a biodegradable cellulose (CMC) packaging film. Ag-MOF@CMC film provides advantages over plastic films that use a lot of energy, such as its high mechanical strength, excellent water insulation, and ecologically friendly preparation. Strawberries packaged in commercial PE cling film, strawberries packaged in CMC film, and unpackaged strawberries all displayed mildew and breakdown on the fifth day following packing. Strawberries wrapped with Ag-MOF@CMC film, however, showed no appreciable alterations. The experiment investigated the bacteriostatic effect of the material against *E. coli*, *S. aureus*, and *Aspergillus niger* using the disc method. The continuous release of silver ions in Ag-MOF@CMC had the best bacteriostatic effect when compared to conventional PE cling film, according to the data. Zhang et al. [83] prepared Ag-MOFs@CMFP by using CMFP as the substrate and homobenzoic acid as the ligand of silver nitrate. The substance had both Ag-MOFs and CMFP that were firmly bonded together. The release of silver ions from the Ag-MOFs permeated the bacteria, inhibiting their development. It successfully extended the freshness of fruits with a high antibacterial rate of 99.9% against *E. coli* and other bacteria. Therefore, Ag-MOF has been prepared using silver ions with organic ligands [84,85] and combined with other materials to provide more options for applications in the field of fruit and vegetable preservation [86,87].

### 4.2. UV-Resistant Applications

UV radiation causes photochemical reactions in fruits and vegetables, leading to a reduction in color, texture, flavor, and nutritional quality [88]. By using silver nanocomposites, it is possible to protect food from photo-oxidation and thus extend its shelf life. Elham et al. [89] prepared UV-resistant composite films using gelatin as a matrix with riboflavin, essential oils (EOs), and Ag NPs and evaluated the UV-blocking properties of the films at a wavelength of 200 nm using UV-visible spectrophotometry. The results showed that the UV transmittance of the composite film was about 2–7%, and it had strong UV resistance. Feng et al. [90] synthesized a C-Ag@PVA/CS composite film for blueberry preservation by preparing Ag NPs from spent barley hulls and using PVA/CS as a carrier. Measurements of the films by XRD revealed a decrease in the peak strength of the C-Ag@PVA/CS composites compared to the PVA/CS composites, suggesting that the Ag NPs may be encapsulated within the composites. Due to the higher stability of Ag NPs, TGA analysis revealed that the thermal stability of the films was improved. Meanwhile, with the increase of C-Ag content, the film gradually became black, and the black film kept the food away from light and reduced the photosynthesis of leafy vegetables, thus prolonging the storage time. With both organic and inorganic components, MOFs have the advantages of good biocompatibility, chemical stability, thermal stability, and optical stability [60,91]. Chen et al. [92] synthesized Ag-MOF in situ on carboxymethyl cellulose filter paper (CMFP) using 2-methylimidazole as a ligand, and this approach improved the UV resistance of CMFP. In the wavelength range of 250–400 nm, the UV transmittance of CMFP was 2.6–20.3%, while that of Ag-MOF/CMFP was only 0.09–1.4%. In addition, preservation tests were conducted with tomatoes and peaches. The results showed that both control and CMFP-packed tomatoes and peaches had developed mold and rot after 6 days. However, the fruits packed in Ag-MOF/CMFP composites maintained their good appearance without significant decay.

### 4.3. Moisture-Resistant Applications

Moisture provides conditions for the growth of bacteria and fungi, so the treatment of fruits and vegetables to block water vapor can effectively extend the shelf life of fruits and vegetables [93]. The use of materials that block water vapor is an option to obtain safer and healthier food [94].

Gelatin is widely used as a base for preservation films, but films with gelatin as a base have high hydrophilicity, which is not beneficial for food preservation. Sarmast et al. incorporated EOs and Ag NPs in gelatin-riboflavin-based films using gamma irradiation [89]. The crosslinked gelatin inhibits the formation of hydrogen bonds, and its dense structure slows down the rate of water absorption and migration, reducing the water vapor transmission rate of the membrane. Ag NPs in combination with EOs were synergistically antimicrobial, which significantly increased the bacterial inhibition rate of the active film. Thus, films that allowed for an extended shelf life were developed, enhanced by their physicochemical properties. Satwik et al. prepared soybean isolate protein composite films using Ag NPs, TA, and kaolin nanotubes [95]. The addition of nanocomposites improved the solubility of the composite film in water, with the water vapor permeability reduced by 1.28–1.30 times, which enhanced the waterproofing properties of the film and consequently improved the freshness preservation effect.

### 4.4. Antioxidant Applications

Oxygen is one of the main causes of food spoilage and quality changes. Fruits and vegetables will undergo an oxidizing reaction when exposed to air because oxygen will react with the nutrients in the produce. It causes oxidative degradation in fruits and vegetables, which lowers their nutritional content and flavor and may even produce toxic compounds [96]. However, the amount of oxygen that fruits and vegetables come into contact with may be efficiently decreased by using food packaging materials and technologies that possess antioxidant qualities. Additionally, these packaging materials can help preserve the freshness and quality of food, as well as stop or postpone the oxidation reaction of fruits and vegetables. Hayat et al. [97] synthesized chitosan-silver oxide nanocomposites using a one-step sol-gel method. The mass percentage of ABTS free radicals that were scavenged rose to 49.91% when the mass percentage of silver oxide nanoparticles was 15%. It is a potential material for developing antioxidant food packaging.

Because Ag-MOF has a double bond and a carboxyl group that may trap free radicals, it can break the cycle of free radical reactions. Therefore, Ag-MOF-containing membranes can also have better antioxidant activity. Zhang et al. [98] prepared a composite food packaging film with multiple functions that combined the properties of starch, PVA, CS, and Ag-MOF.

### 4.5. Gas Conditioning Applications

In the storage, transportation, and marketing of fruits and vegetables, reasonable control of the gas environment is very important to maintain their freshness and extend their shelf life [99]. Sumaira et al. [100] successfully prepared a spray solution for mango preservation using glucose oxidase (GOx)-modified Ag NPs. The results showed that fungal growth could be effectively inhibited with the spray solution, and it was able to prolong the preservation period of mango up to nine days, whereas the control group showed fungal spots on the third day. The preservation mechanism was a multifunctional and synergistic effect of the materials used. First, Ag NPs/GOx can be used as a deoxidizer in addition to an antimicrobial agent. Secondly, the biocatalytic activity of Ag NPs/GOx produces a layer of H_2_O_2_ on the surface of the samples, inhibiting the respiratory process and water loss of the fruits, further enhancing the freshness effect. Ethylene, a plant growth regulator, accelerates the ripening of fruits and vegetables, and the shelf life of fruits and vegetables can be extended by controlling ethylene levels. Thu Hoai et al. [101] demonstrated that loading Ag on TiO_2_ improves the photocatalytic performance of TiO_2_ for more efficient ethylene removal by comparing the energy gaps of TiO_2_ and Ag/TiO_2_. This type of preservation has an important potential for application in the field of fruit and vegetable storage.

Some of the recent research results on fruit and vegetable preservation containing silver nanocomposites are listed in Table 1.

## 5. Conclusions

Silver nanocomposites have excellent antimicrobial properties, adjustable structure and morphology, good biocompatibility, and controllable release of silver ions. These advantages enable silver nanocomposites to achieve multifunctional food preservation and better meet consumer demand for food quality and safety. However, further studies are needed on the biodegradability, potential toxicity, and mechanisms of interaction with food of silver nanocomposites in order to ensure their safety and sustainability. These investigations may include both in vivo and in vitro research. The aim is to assess the absorption, metabolism, and excretion of silver nanocomposites in the human body. In addition, studies on the environmental impacts of silver nanocomposites are needed to minimize potential harm to the environment, including their distribution and bioaccumulation effects in soil, water, and ecosystems. In addition, the antimicrobial activity of silver nanomaterials may gradually decrease over time due to repeated use. This situation will be obvious in the long-term storage condition. Reduced preservation capacity may cause long-lasting shelf life to be affected because microorganisms may gradually adapt and develop resistance to silver nanomaterials. Therefore, periodic replacement or supplementation with other preservation methods needs to be considered, especially when using silver nanomaterials for fruit and vegetable preservation for longer periods.

While silver nanocomposites show great potential for fruit and vegetable preservation, additional research and evaluation are needed to ensure their sustainability and safety, as well as the viability and reliability of their broad application. There are many methods for the preparation of silver nanocomposites at present. However, we need to be aware of the environmental impact of nanomaterials. Environmentally friendly silver nanocomposite preparation techniques can be developed, which can reduce the potential environmental impact of silver nanocomposites on the environment. Because silver nanocomposites may not retain their ability to retain freshness over time, new material combinations or technologies could be utilized to extend the period for which their antimicrobial properties are maintained. This ensures the stability of the long-term freshness effect.

**Figure 7:**
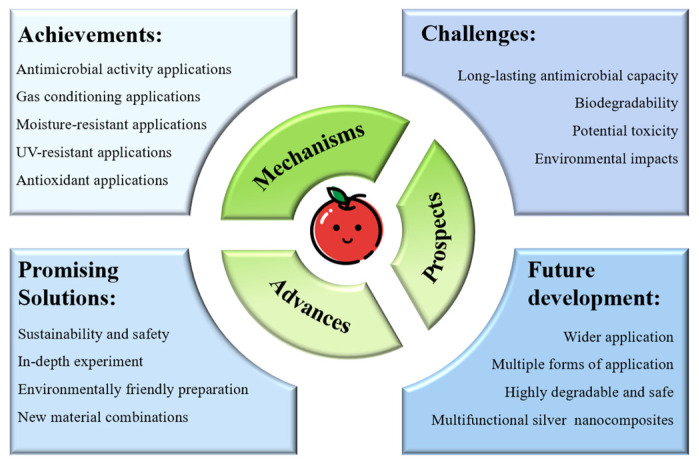
The development of silver nanomaterials in the field of fruit and vegetable preservation.

Silver nanocomposites have the prospect of wide application in the field of fruit and vegetable preservation. Research on silver nanocomposites will tend to be multifunctional in the future. The nanomaterials prepared in the future will not only have excellent antimicrobial activity but will also be able to influence the ripening rate of fruits and vegetables and slow down the spoilage process. In addition to conventionally used fruit and vegetable preservation applications, silver nanocomposites may also be used in the future as fruit and vegetable additives to regulate the quality of fruits and vegetables (see Figure 7).

## Figures and Tables

**Table 1:** Recent application of silver nanocomposites in fruit and vegetable preservation.

Type of Ag NPs	Preservation Principle	Antibacterial Species	Application	References
PBST/MgO/Ag complex film	bacteriostatic, waterproof	*E. coli*, *S. aureus*	tomato preservation	[102]
PVA/SA/Ag–TiO_2_ NPs complex film	bacteriostatic	*E. coli*, *S. aureus*	food packaging	[38]
agar/Ag-carbon dots complex film	bacteriostatic, UV resistant	*E. coli*, *S. aureus*	tomato preservation	[103]
Ag/PMMA complex film	bacteriostatic, waterproof, UV resistant	*S. aureus*	food packaging	[39]
PVA/SA/Ag–Se NPs complex film	bacteriostatic, UV resistant	*E. coli*, *S. aureus*	food packaging	[104]
PVA/CS/GO/Ag NPs complex film	bacteriostatic	*E. coli*, *S. aureus*	food packaging	[105]
Nanocellulose/arabinoxylan/ Ag NPs complex film	bacteriostatic, waterproof, UV resistant	*Shigella flexneri*, *P. aeruginosa*, *aspergillus brasiliensis*	food packaging	[106]
Ag/TiO_2_ powder	bacteriostatic, controlled ethylene	*E. coli*, *S. aureus*	fruit preservation	[101]
CS-Ag/TiO_2_ fiber complex film	bacteriostatic, waterproof, UV resistant	*E. coli*	pepper, and banana preservation	[29]
Ag@PVA/CS complex film	bacteriostatic, waterproof, UV resistant, antioxidative	*E. coli*, *S. aureus*, *bacillus subtilis*, *P. aeruginosa*	blueberry preservation	[90]
Ag NPs/montmorillonite powder	bacteriostatic	*E. coli*, *S. aureus*, *salmonella*, *P. aeruginosa*, *listeria monocytogenes*	food packaging	[30]
Ag/PMMA complex film	bacteriostatic, waterproof, UV resistant	*S. aureus*	food packaging	[39]
GOx modified Ag NPs liquid	bacteriostatic, oxygen removal		mango preservation	[100]
Ag NPs synthesized by Agaricus bisporus	bacteriostatic	*E. coli*, *S. aureus*	food packaging	[57]
CS/Ag NPs complex film	bacteriostatic, waterproof, UV resistant, antioxidative	*E. coli*, *S. aureus*	apple preservation	[16]
Ag NPs- grape peel extract/PVA complex film	bacteriostatic, antioxidative, maintain moisture	*E. coli*, *B. subtilis*	food packaging	[107]
Ag NPs synthesized from pomegranate extract	bacteriostatic, antioxidative, antiviral	*E. coli*, *S. aureus*	orange preservation	[52]
carbamate starch/lignite/cellulose microfibril/Ag NPs masking liquid	bacteriostatic, waterproof, UV resistant, antioxidative, blocking oxygen	*E. coli*, *S. aureus*	tomato preservation	[108]
soybean polysaccharide /Ag NPs Janus film	bacteriostatic, waterproof, UV resistant, antioxidative	*E. coli*, *S. aureus*	grape preservation	[109]
cellulose/starch/Ag NPs complex film	UV resistant		food packaging	[110]
PVA/cassava starch/CS/Ag NPs complex film	bacteriostatic	*Bacillus perfringens*, *S. aureus*, *B. subtilis*, *aspergillus flavus*	tomato, orange, coffee bean preservation	[111]
PVA/beta-cyclodextrin/Gallic acid functionalized Ag NPs complex film	bacteriostatic, waterproof, UV resistant, antioxidative	*E. coli*, *S. aureus*, *A. flavus*, *candida albicans*	food packaging	[112]
SA/guar gum/anthocyanin extract of wolfberry /Ag NPs/BO Pickering emulsion-complex film	bacteriostatic, waterproof, UV resistant, antioxidative, blocking oxygen	*E. coli*, *S. aureus*	mushroom preservation	[113]
Ag/ZnO NPs powder	bacteriostatic, maintains moisture, inhibiting metabolism		peach preservation	[114]
Ag-MOF@CMC		*E. coli*, *S. aureus*, *A. niger*	strawberry preservation	[82]
Ag-MOF/CMFP	bacteriostatic, UV resistant	*E. coli*, *S. aureus*	tomato, peach preservation	[92]
Ag-MOFs@CS suspension liquid	bacteriostatic	*E. coli*, *S. aureus*	strawberry, pitaya preservation	[115]
PVA/starch/CS/Ag@MOF complex film	bacteriostatic, waterproof, UV resistant, antioxidative, isolation oil	*E. coli*, *S. aureus*	food packaging	[98]
Ag-MOFs@CMFP	bacteriostatic, humidity response, quality monitoring	*E. coli*, *S. aureus*, *A. niger*	strawberry, blueberry, plum preservation	[83]

## Data Availability

Data sharing is not applicable for this review.
